# Achieving optimal best practice: An inquiry into its nature and characteristics

**DOI:** 10.1371/journal.pone.0215732

**Published:** 2019-04-25

**Authors:** Huy P. Phan, Bing H. Ngu, Hui-Wen Wang, Jen-Hwa Shih, Sheng-Ying Shi, Ruey-Yih Lin

**Affiliations:** 1 School of Education, University of New England, Armidale, Australia; 2 Department of Asian Philosophy and Eastern Studies, Huafan University, New Taipei City, Taiwan; 3 Department of Buddhist Studies, Huafan University, New Taipei City, Taiwan; 4 Department of Industrial Engineering and Management Information, Huafan University, New Taipei City, Taiwan; Kyoto University, JAPAN

## Abstract

The study of *optimal best practice* within the context of academia has produced both empirical and theoretical contributions. Optimal best practice, also coined as optimal functioning, is concerned with a person’s personal best–that is, “what is the best that I can do for this academic subject?” Research in the social sciences has, to date, explored different types of optimal best–physical, cognitive, emotional, and social. What is of considerable interest, as a related matter, is a question of how a person reaches and experiences a level of optimal best practice. Recent research development, for example, has explored various conceptualizations of optimal best practice–for example, one distinctive theoretical model, the Framework of Achievement Bests [1, 2], makes a concerted effort to explore the *underlying process of optimization*–that is, in this case, how an optimal level of best practice is achieved.

The present study, as detailed below, investigates via means of non-experimental data a theoretical model pertaining to the achievement of optimal best practice. This examination, we postulate, would enable us to add clarity and provide additional theoretical insights the operational nature of the process of optimization. The operational nature of optimization, as described in our recent research [1, 3], emphasizes three major tenets: (i) the main sources of a person’s optimal best practice, (ii) the potential ‘optimizing’ influences of three comparable agencies on the achievement of optimal best practice (i.e., personal resolve, social relationship, and personal self-efficacy), and (iii) the impact of optimal best practice on future adaptive outcomes (i.e., academic striving and personal well-being). We explored this topic via means of the use of a non-experimental, correlational design with participants drawn from Taiwanese university students (*N* = 1010). Structural equation modelling (SEM) produced evidence, which empirically supported existing research [1, 3] and substantiated our knowledge of the concept of optimal best practice.

Evidence established from the present study has also assisted us to identify one pervasive issue, which we call for further research development–namely, to consider, design, and develop an appropriate methodological approach that would enable researchers to accurately measure and assess the process of optimization. Finally, in terms of teaching and learning, we acknowledge that our research investigation has provided some insights into potential educational practices for implementation.

## Introduction: Achieving optimal best practice

One line of inquiry in Educational Psychology that has been researched by a number of scholars is related to a person’s *optimal best practice* in a subject matter [4–6]. “What is my personal best?” is a question that a person often makes queries of. Optimal best practice, also known as optimal functioning, may consist of different types [7], for example: optimal cognitive functioning (e.g., a student’s exceptional result in his half-yearly exam in mathematics), optimal physical functioning (e.g., a football player’s accomplishment of scoring of 50 goals in 2017/2018 season), and optimal emotional functioning (e.g., a person’s state of happiness). Personal experience of optimal best practice is a central feat of human agency, and reflects the tenets of the *paradigm of positive psychology* [8, 9]. Optimal best practice, in fact, is an antithesis of maladaptive functioning (e.g., a state of disengagement) [10] and engagement of negative outcomes.

Educationally, one area of research that is noteworthy for development is related to a student’s achievement of optimal best practice–that is, how does a student experience a state of optimal best practice in, say, mathematics? As educators, we need to consider different types of in-class interventions and/or school-based programs that could facilitate, promote, and foster personal best. This consideration has led educators and researchers to propose alternatives and different pathways that could effectively explain optimal best practice. One major line of development is related to researchers’ analytical discussions of theorizations and methodological conceptualizations of optimal best practice [5, 11–13]. Fraillon’s [11] discussion preliminary paper of *subjective well-being* in school contexts introduces the term ‘human optimization’. Relatively brief in its description, Fraillon [11] defines optimization as the difference between a person’s *current level of best functioning* (e.g., denoted as ‘L_1_’) and his/her *optimal level of best functioning* (e.g., denoted as ‘L_2_’). Capitalizing on this definition, Phan, Ngu, and Williams [2] proposed that a person’s *realistic achievement best* (i.e., equivalent to current level of best functioning, L_1_) could also serve as a source of his/her *optimal achievement best* (i.e., equivalent to optimal level of best functioning, L_2_). In a follow-up discussion, Phan, Ngu, and Yeung [1] introduced a more detailed conceptualization of optimization, coined as the *Framework of Achievement Bests*.

**Fig 1:**
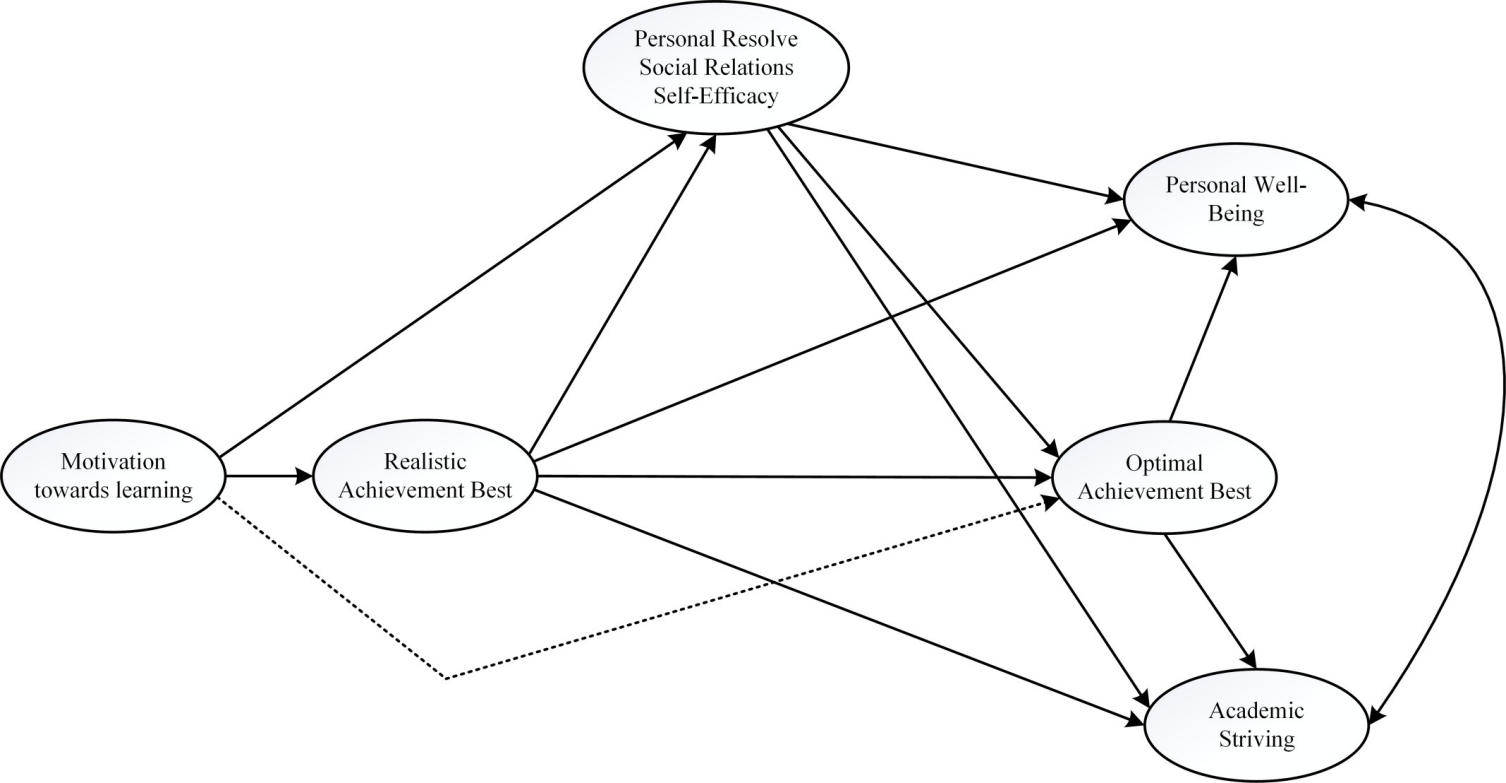
Conceptual model for research development.

Overall then, from this brief introduction, we propose a theoretical model of optimal best practice for examination, which in this case involves the use of correlational, non-experimental data. Our theoretical model, as shown in Fig 1, reflects the direct influences of different *sources* (i.e., denoted as ‘S’) on optimal best practice, and the *subsequent effect* of optimal best practice on different types of adaptive outcomes (i.e., denoted as ‘O’). Importantly, our theoretical model attempts to address one fundamental question that, to date, remains elusive: how can we derive understanding of optimal best practice and its positive effect by means of using correlational data without any experimental treatment? Our address of the mentioned question, in this case, explores four major propositions: (i) the direct impact of a person’s realistic best practice on his/her optimal best practice (i.e., L_1_ → L_2_), (ii) psychological variables (e.g., the concept of ‘motivation towards learning’) that could operate as direct sources of optimal best practice (i.e., S → L_2_), (iii) psychological variables that could act as mediators (i.e., the concepts of ‘social relationships’, ‘self-efficacy’, and ‘personal resolve’) between optimal best practice and its direct sources, and (iv) the subsequent positive effect of optimal best practice on ‘academic striving’ and ‘personal well-being’ as adaptive outcomes. Overall then, we reason that the use of *structural equation modelling* (SEM) techniques [14, 15] could help to elucidate the nature of optimal best practice, especially in terms of its makeup and subsequent effect (e.g., L_1_ → mediator → L_2_). Our proposition, discussed in detailed below, acknowledges the potency of different ‘psychological agencies’ that could improve L_1_ to L_2_.

## A Theoretical-conceptual model of optimal best practice

Optimal best practice is an interesting theoretical concept that focuses on the ‘positive nature’ and personal growth of individuals and society. Optimal best practice reflects a person’s resilience, inner strengths, virtues, and aspirational outlooks. An analysis of the literature in the areas of Education, health and mental functioning, and subject well-being indicates relatively consistency in terms of scope and definition of optimal best practice–for example, in brief, optimal best practice has been defined as the following: “maximizing one’s potential” [16, 17], “pursuit of excellence in physical, mental, emotional, and spiritual realm” [18], “an active process of fulfilment” [19], “living and working effectively” [20], “living fully in the natural community” [21], “resilience and successful community participation” [22], “holistic, positive emotions” [23], “positive emotions, life satisfaction, and absence of negative emotions” [24], “positive feelings and positive psychosocial functioning” [25], “resilience, satisfaction, and maximizing one’s potential” [26], “positive feelings and life satisfaction” [27], “positive state and satisfaction of needs” [28], and “resilience and maximizing one’s potential” [29].

From the preceding section, it is clear and evident that optimal best practice is concerned with positive experience and the maximization of a person’s potential. Optimal best practice, contextualized within the context of academic learning (e.g., optimal best in Literature), may indicate a person’s level of exceptionality of performance in different subject matters [4]. “This is the best that I can do …..” is a personal statement that often resonates and, in many cases, motivates a student to achieve exceeding performances at school or university. Indeed, as recent research development has shown [6, 7], optimal best practice is beneficial and may result in the achievement of different adaptive outcomes. *Why study optimal best practice*? Optimal best practice is a positive and non-deficit facet of cognition and human behaviour. Rather than focusing on the remedy and preventive measures of maladaptive academic functioning, optimal best practice instead centres on proactive engagement, persistence, and effort expenditure.

At the same time, of course, aside from academic contexts, researchers have also focused on other theoretical models and constructs, which are similar to the concept of optimal best practice. For example, the recent work of Diener and his colleagues has explored the theoretical concept of *thriving* [30, 31], which is defined as a person’s “state of positive functioning at its fullest range–mentally, physically, and socially” [30]. A similar concept that has been studied by researchers is that of *a state of flourishing* [32, 33, 34], in this case refers “to the experience of life going well” for a person [33]. Csíkszentmihályi’s theory of *flow* [13, 35], likewise, emphasizes the importance of a person’s state of absorption, intense concentration, and enjoyment of engagement of a particular task–for example, a child’s intense concentration and experience of ‘flow’ as he/she attempts to solve the rubric cube. This development of thriving, flourishing, and flow interestingly coincides with other theorists’ conceptualizations of positive subjective well-being in life [36, 37]. *Self-determination theory*, based on earlier humanistic psychology theories [38, 39], offers an in-depth account of notable universal psychological needs that a person may consider for the development of his/her well-being, such as the need for competence, relatedness, and self-acceptance.

Indeed, from the perspective of positive education and psychology, the concepts of flow, flourishing, and thriving are of significance for their emphasis on individual growth, the enrichment of personal experience in life, and ‘feel-good’ emotions (e.g., a state of happiness). Optimal best practice, we contend, may complement and enhance our understanding of the operational nature and functioning of the mentioned theoretical concepts (e.g., flow). As existing theorizations postulate, the ‘essence’ of optimal best practice entails a uni-directional progress or ‘movement’ from one level (e.g., novice) to that of another level (e.g., expert). As such, it can be reasoned and argued that in this instance, ‘personal experience’ of optimal best practice in a subject matter may reflect a person’s enriched state of flourishing–coined it from another positioning then, an experience that “life is going well” reflects the achievement of optimal best. More importantly, of course, we reason that the nature of optimal best practice in itself is positive, enriching, and motivational.

In a similar vein, in academic contexts, the study of optimal best practice has educational merits and may provide in-depth understanding of students’ motivational beliefs and learning patterns [6, 40]. Confidence, self-determination, and situational interest, for example, may serve as informational sources in the formation of optimal best practice [1]. A student’s determination and decisiveness to gain mastery, in this analysis, may motivate and compel her to achieve optimal best. At the same time too, from existing research development, optimal best practice may operate to positively influence different types of educational outcomes. For example, in a recent longitudinal study, Liem et al. [6] found that personal best sustained its positive effect on different achievement-related outcomes, for example: the effect of T_1_ personal best on T_1_ deep learning (β = .72, p < .05), and the effect of T_2_ personal best on T_2_ deep learning (β = .45, p < .05). In a cross-sectional study, which involved Taiwanese university students, likewise, we note that optimal best positively influenced three comparable outcomes: motivation towards learning (β = .43, *p* < .001), academic liking experience (β = .25, *p* < .001), and personal interest (β = .57, *p* < .001)[7].

One area of research, currently underdeveloped, is that of a specific underlying process that could govern and explain a person’s achievement of optimal best practice. Our assessment of the literature indicates that a number of theoretical models and perspectives, which are worthy of development. Vygotsky’s [41] *sociocultural theory of cognitive theory*, interestingly, emphasizes the importance of *instructional guidance* and the provision of *psychological tools* and *cultural artefacts* to ‘scaffold’ a person’s cognitive development. Piaget’s [42, 43] *theory of personal constructivism*, likewise, places emphasis on the importance of a person’s *self-discovery* and *self-exploration* in order to resolve his/her ‘cognitive disequilibrium’. More recently, of course, other researchers have considered a more definitive positioning for development. Both Fraillon’s [11] discussion paper of subjective well-being and Phan et al.’s [1, 2] theorization of optimal functioning have made reference to an important psychological process, known as *optimization*. The ‘enactment’ of optimization, according to the authors, would then assist and facilitate in the achievement of optimal best practice.

**Fig 2:**
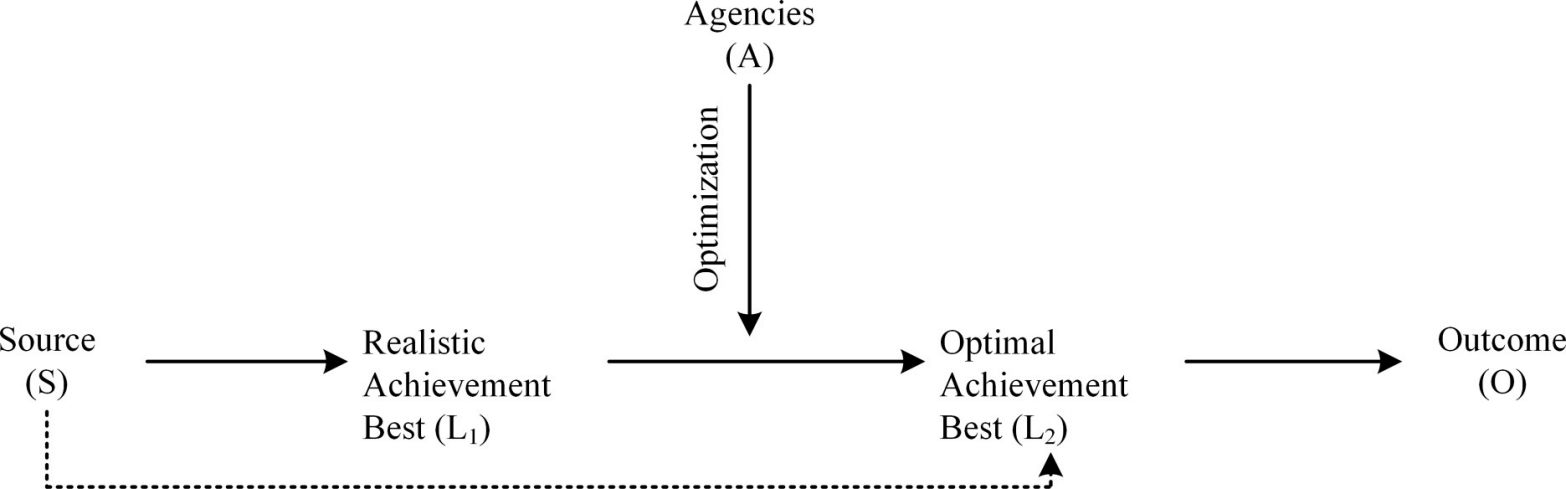
The importance of optimal best practice.

Indeed, from the preceding sections, how a person reaches optimal best practice is a focus for consideration. Our undertaking, reflecting substantive theoretical and methodological contributions, premises a sequenced account of how a person could achieve optimal best practice (i.e., denoted as ‘L_2_’) from his/her realistic best practice (i.e., denoted as ‘L_1_’). Phan and colleagues [1, 44] recently provided a detailed theoretical model of optimal best practice and the process of optimization, which we have surmised in Fig 2 for understanding.

Phan et al.’s [1, 44] theorization, as indicated here in Fig 2, posits that realistic best practice as well as other variables could operate as direct sources of optimal best practice (i.e., S → L_2_). Importantly however, according to the authors, there are educational, psychological, and psychosocial variables, termed as ‘optimizing agents’ (i.e., denoted as ‘A’), that could assist in the achievement of L_2_ from L_1_. This complex process of optimization, which we visually depict as ‘↓’, is extremely difficult to measure, assess, and validate. Our present undertaking adopts a similar, but more simple theoretical approach, as shown in Fig 1 in the earlier section, whereby we propose three main issues for discussion: sources of information, the importance of L_1_-L_2_, and the positive effect of L_2_.

### Sources of information

In their original writing, Phan et al. [1] proposed that both L_1_ and L_2_ do not simply exist in isolation, but rather subsume within a holistic system of change whereby potential sources of information (i.e., denoted as ‘S’) could operate to influence the indication of L_1_ (i.e., S → L_1_). “What I am capable of at present ….” may largely derive from a student’s existing track record of accomplishments. In a similar vein, positive experience of motivation may serve to ‘energize’ a student’s learning experience by instilling confidence, resolute, and personal belief of his actual accomplishments. In the present study, we consider one notable source of information that could determine a student’s L_1_, namely, his/her internal state of *motivation towards learning* [45]. Motivation towards learning, in this case, reflects a student’s level of positive motivational beliefs to succeed in his learning for different subject matters. In the context of schooling, as the extensive literature suggests, a heightened state of motivation is analogously associated with academic performance. Proactive engagement and improved academic performance outcomes, for example, may reflect a student’s high level of motivation (e.g., intrinsic) for learning [46–48]. In a similar vein, from an alternative point of view, a positive state of motivation may correspondingly yield an appropriate level of engagement and/or academic performance. Hence, from this theoretical tenet, we posit that a high level of positive motivational beliefs (e.g., “I work hard for all academic subjects to get good results”) is likely to associate with a strong indication of L_1_, whereas a high level of negative motivational beliefs (e.g., “I think that I rarely do my best at school”) is more in line with an indication of low L_1_.

### The importance of L_1_-L_2_ difference and the impact of psychological agencies

Aside from the formation of L_1_, a pervasive issue for consideration is how then does L_1_ relate to L_2_ (i.e., L_1_-L_2_ difference) and, more importantly, how does a person achieve L_2_? In a recent non-experimental study, via means of correlational analyses, we found that L_1_ exerted a positive effect on L_2_ (β = .29, *p* < .001). This finding (i.e., L_1_ → L_2_) is insightful and coincides with Bandura’s [49] *social cognitive theory* of the significance and relevance of a person’s enactive learning experience–that is, a person’s prior and existing accomplishments forming a potent source of information in the prediction of his/her motivational beliefs and future outcomes.

In addition to the positive impact of L_1_, Phan et al.’s [1] recent conceptualization of optimization also stipulates a notable theoretical tenet–namely there are psychological agencies that could operate in a dynamic, mediating system to facilitate the improvement of L_1_ from L_2_. In this dynamic, mediating system, a psychological agency may serve to ‘energize’ a person’s state of cognitive and motivational processes, which then help to improve his/her level of best practice. Proceeding with this testament, we propose three comparable psychological agencies for examination:

1. *Personal resolve*, which is defined as a person’s “internal state of decisiveness and resolute to strive for optimal achievement best in an optimistic manner” [50]. This definition places emphasis on a person’s “internal state of decisiveness and resolute to strive for optimal achievement best in an optimistic manner” (p. 415). According to Phan, Ngu, and Alrashidi [50], experience of personal resolve in school contexts may assist a student to overcome different obstacles that may arise. Personal resolve, in this sense, is concerned a student’s strong sense of self-determination to stay on task without any indication of uncertainty, mental weakness, and/or indecisiveness. From this understanding, the purposive nature of personal resolve may facilitate and motivate a student’s quest to achieve a course of action in a positive and decisive manner.
  An important question for us to consider is whether and/or to what extent the deliberate nature of personal resolve would mediate the positive effect of L_1_ onto L_2_. We contend that the positive nature of personal resolve, which we liken it to a state of resilience [51, 52], may help a student remains focused and to stay on task, serving as a source of optimal best practice. Personal resolute differs from other comparable constructs for its emphasis on a person’s determination, mental strength, and a ‘unchanging mindset’ to achieve a specific course of action, regardless of shortcomings, obstacles, and/or incorrect results that may arise. From this theoretical positioning, we argue that personal resolve is likely to produce positive yields on both short-term and long-term outcomes.
  To date, from our examination, a few research studies have explored the operational nature of personal resolve in educational contexts. For example, in a longitudinal study, Phan, Ngu, and Alrashidi [50] found that personal resolve exerted a temporally-displaced effect on contextualized self-efficacy (e.g., T_2_ personal resolve → T_3_ task-specific self-efficacy, β = .14, *p* < .05). A recent research undertaking [53], which involved the use of cross-sectional data, we noted that personal resolve positively influenced academic achievement (β = .16, *p* < .05). This evidence, overall, has provided a valid basis to incorporate this psychological concept into the study of optimal best practice.
2. *Social relationships*, which may consist of different types (e.g., a teacher-student relationship)[54], have also been noted to play a central role in helping students adjust to the academic learning processes [55, 56]. Proactive social relationships with people at school, according to Van Damme et al. [45], may have profound influences on a student’s subjective well-being and cognitive learning experiences. This premise regarding the positive impact of social relationships at school, as Roorda et al. [56] explain, arises from the fact that others (e.g., teachers) in the school system may provide an emotional security-base by which students could then feel safe to learn, explore, etc. There is empirical research that has reported consistent evidence pertaining to the positive effect of social relationships at school on academic performance and other achievement-related outcomes [10, 57, 58]. Being able to socially relate to others, in this analysis, may enable a student to reach out and seek learning, moral, social, and/or emotional support that, in turn, could provide a basis for the achievement of optimal best practice.
  An important question to consider then, from the preceding section, is whether and/or to what extent social relationship could assist in the facilitation of optimal best practice. This consideration places emphasis on peers, friends, teachers, and others who could centrally provide scholarly guidance, emotional and social support, and/or normative evaluation information for motivation purposes. A student may seek out academic assistance from a capable peer to help him/her understand a subject matter. A peer who shares comparable interests and similar academic ambitions, likewise, may convey relevant information for social comparison and personal reference purposes, which could then assist another student in his/her quest to achieve optimal best practice. For example, a student may observe his/her best friend’s personal ambition to achieve optimal best in mathematics, and in turn uses this vicarious learning as a source of motivation [49, 59]. From the paradigm of positive psychology [8, 9], a student may often use a typical catch phrase–“If he can achieve top results, so can I….”–to motivate and convince himself/herself. Academically negative social experiences at school (e.g., the inability of a student to get on with others…), in contrast, may also result in detrimental consequences–engagement in antisocial behaviours [60], ineffective study habits, and in the present context the inability of a student to achieve optimal best practice.
3. *Personal self-efficacy*, situated within social cognitive theory [49], is an important non-cognitive construct that may serve to predict different types of adaptive outcomes. Self-efficacy, according to Bandura [49], is defined as “beliefs in one’s capabilities to organize and execute the courses of action required to produce given attainments” (p. 3). Self-efficacy, in this sense, is not concerned with a person’s actual ability but rather a self-judgment of perceived competence–for example, regardless of my existing capability, do I believe that I have the confidence to solve…. ? Self-efficacy is a contextualized type of self-belief, differing from self-esteem and self-concept, which are global and domain-specific.
  Personal self-efficacy [49], as prior research has shown, is a potent predictor and mediator of future outcomes [61, 62]. Self-efficacy, according to Bandura [49], is effective as it governs a person’s choices in life (e.g., choosing an appropriate career pathway), mobilizes his/her effort expenditure and state of persistence (e.g., persisting a course of action despite personal experience of difficulties), and self-regulates appropriate physiological and emotional responses (e.g., lower a high level of anxiety). Inefficacy, in contrast, may serve to weaken a person’s state of persistence and effort expenditure, resulting in the undermining of performance outcomes. In academic contexts and in the area of student motivation, it is recognized that academic self-efficacy helps to explain and predict a student’s learning experience and achievement in different subject areas [50, 63, 64]. A high level of self-efficacy is analogously associated with an improvement in academic performance, whereas a low level of self-efficacy is more aligned to weakened performance outcomes. As prior research has shown, likewise, personal self-efficacy for academic learning may also mediate different types of psychosocial factors and cognitive and motivational processes on future educational outcomes [63, 64, 65, 66]. In this analysis, as a number of correlational studies have affirmed, different psychosocial, cognitive, and motivational variables may indirectly influence academic performance and other achievement-related outcomes, via personal self-efficacy.
  We purport that, overall, there is empirical support for the rationalization of the inclusion of academic self-efficacy as an optimizing agent of optimal best practice. This postulation considers, in particular, the importance of the mediating potential of self-efficacy for academic learning. In a recent longitudinal study, for example, Martin et al. [63] noted that self-efficacy mediated the positive effect of academic buoyancy for learning at T_1_ on itself at T_2_. In a similar longitudinal research, Phan et al. [50] found that task-specific self-efficacy at T_3_ mediated the positive effect of effective functioning at T_2_ on school experience at T_4_. From this consideration of existing evidence, we query whether and/or the extent to which L_1_ could indirectly influence L_2_, as mediated by self-efficacy for academic learning. Validating the direct positive influence of self-efficacy on L_2_, similarly, has both theoretical and practical merits–a heightened level of self-efficacy, in this case, may operate as a source of motivation, encouraging and compelling a student to persist and strive for exceptionality.

### The positive effect of L_2_

Phan et al.’s [1] conceptualization of optimal best practice, concurring with existing research development [6, 7], postulates that L_2_ could actually serve as an indicator and predictor of future educational outcomes (i.e., denoted as ‘O’). This postulation (i.e., L_2_ → O) contends that personal experience of optimal best practice could, indeed, act as a ‘motivational basis’ for further educational acquisition. A student’s optimal best practice is postulated to act in tandem with the positive impact of an agency (i.e., denoted as ‘↓’ representation), which may then encourage and predict future accomplishments. There is evidence, at present, to empirically support the postulation regarding the predictive role of optimal best practice. In a longitudinal study, Liem et al. [6] reported that a student’s personal best positively predicted a number of achievement-related outcomes, for example: academic flow in learning (e.g., T_1_ personal best → T_1_ academic flow, β = .74, *p* < .05), deep learning (e.g., T_1_ personal best → T_1_ deep learning, β = .72, *p* < .05), and teacher social relationship T_1_ personal best → T_1_ teacher social relationship, β = .61, *p* < .05). In another similar longitudinal research project, Martin and Liem [67] found a number of interesting patterns for personal best–for example, the positive effect of T_1_ personal best on T_2_ educational aspirations (β = .07, *p* < .01), and the positive effect of T_1_ personal best on T_2_ enjoyment of school (β = .04, *p* < .01). Notwithstanding the limitation of cross-sectional data, we [7] noted that optimal best practice positively influenced different types of adaptive outcomes–for example (e.g., optimal best practice → personal interest in learning, β = .57, *p* < .001).

From the preceding sections, taking into consideration the clear and consistent evidence from both cross-sectional and longitudinal research, we postulate that L_2_ would positively predict two comparable adaptive outcomes, namely: (i) *academic striving*, which is defined as a student’s effortful attempt to seek out a realistic and/or an ambitious endeavour for accomplishment [40], and (ii) *personal well-being experience*, which in this case is defined as the extent to which a student enjoys attending school or university for academic purposes [45]. Academic striving, similar to that of personal thriving [30, 31], is a positive construct, providing relevant information into a student’s state of aspiration, contemplation, and motivation to succeed in life. Indication of high academic striving to succeed in mathematics, for example, would correspondingly convey a high level of motivation. Indication of low academic striving, in contrast, would suggest minimal aspiration, contemplation, and motivation. More importantly, from our point of view, a low level of academic striving would connote inclination towards procrastination and personal experience of helplessness.

Personal well-being experience is an important index of successful schooling. In recent years, educators and researchers have come to recognize that academic performance alone is limited and does not provide an accurate account of a student’s academic experience. Some students, for example, may not necessarily achieve high academic results, but yet still enjoy their schooling experiences. This testament emphasizes the fact that schooling, in its totality, may espouse numerous educational indexes [45, 58]. One notable index that has received considerable attention of late is subjective well-being [68, 69].

Overall, we choose to explore both academic striving and personal well-being experience, consequently as a result of their positive nature and comparable characteristics. It would be of interest for us to consider the extent to which L_2_ could positively influence academic striving and/or personal well-being experience. This postulation places emphasis on the potentiality of L_2_ to influence different types of school-based adaptive outcomes. Testament of evidence, in this case, would theoretically advance our understanding and contribute to the characteristics of optimal best [1, 4, 6]. At the same time, of course, a statistically significant influence of optimal best practice would help support existing rationales concerning the inclusion of academic striving and personal well-being. A focus on different educational experiences other than academic performance is poignant, as it connotes the philosophical positioning that successful schooling espouses much more than just performance-based outcomes.

## Significance of the research

Overall then, as a point of summation, the present study is unique for its proposition of an explanatory account of optimal best practice. Understanding of optimal best practice, from our point of view, is similar to Vygotsky’s [41] theoretical tenet of the ZPD and Piaget’s [42] emphasis of personal experience of conflict resolution. Our theoretical model for investigation is significant for its depiction of three major inquiries, namely: the impact of an appropriate source of information on realistic best practice (i.e., S → L_1_), the potential for different psychological variables to operate as mediators and to mediate the effect of a source (e.g., realistic best practice) onto optimal best practice (i.e., L_1_ → mediator → L_2_), and the positive influence of optimal best practice on various adaptive outcomes (i.e., L_2_ → O).

Our focus of examination (e.g., the impact of a psychological agency to mediate the influence of L_1_ onto L_2_), which researchers have not yet explored, is progressive and may provide continuing theoretical insights into the operational nature of optimal best practice–for example, how does a person reach a state of optimal best practice in life? In this sense, accounting for the magnitude of the L_1_ and L_2_ difference (i.e., Δ_(L1-L2)_) is an interesting inquiry for development. Embarking on this research, we recognize that the study of the nature of optimal best practice is not an easy feat. Our proposed non-experimental undertaking, notwithstanding its limitations, has significance for consideration. Notably, in this case, is our main objective to expand on current understanding of optimal best practice. The advancement of our inquiry encompasses methodological and theoretical contributions into the process of human optimization.

## Methods

### Sample and procedure

The study reported in this manuscript was approved by the University of New England's Research Ethics Committee, Number: HE13-230. We verbally sought permission at the onset by asking any participant who did not wish to take part in the study to inform us. This method of verbally seeking participatory consent, which we previously used in a number of our research, was logistically convenient and appropriate given the ages of the participants. A total sample of 1010 undergraduate students (*N* = 405 males, 605 females) from seven universities (i.e., two public universities, five private universities) located in Taipei City and New Taipei City, Taiwan took part in the study. In Taiwan, there are two types of university: (i) private university, which is private and privately funded by the student, himself/herself, and (ii) public university, which is public and, in many cases, more prestigious and competitive. The majority of the participants were from the private universities (*N* = 878). Entry into a public university in Taiwan (e.g., National Taiwan University) is an extremely competitive process, relying on high academic results. Students who do not meet the cut-off threshold into a public university may then proceed onto entry into private universities. Despite her modest size, Taiwan has more than 100 universities and colleges for students to choose from. Our sampling was convenient as it was logistically difficult to seek permission from students in other universities and colleges to take part in the present research study. Aside from this difficulty, limited resources also deterred us from attempting to expand on our data collection.

The participants voluntarily took part in the study, knowing that there were no incentives and that they could withdraw from the study anytime during the course of the data collection process. The questionnaires were administered using a paper-format in lectures and tutorial classes. The questionnaires took approximately 25–30 minutes to complete, and participants were encouraged to ask for clarification at the end, if necessary. The questionnaires consisted of a front-page demographic information sheet, which required the participants to indicate the following: gender (e.g., male), university (e.g., National Taiwan University), department (e.g., Department of Engineering), course of study (e.g., Bachelor of Liberal Arts), age, and study status (e.g., Full-time).

The medium of formal instruction at school and in university is Chinese Mandarin. The questionnaires, originally conceptualized in English, were translated to Chinese Mandarin for the participants. A three-step methodological procedure was undertaken: (i) the questionnaires were first translated from English to Chinese Mandarin by one of the authors and another Ph.D. student at one of the Taiwanese universities (Note: the Ph.D. student also specialized in the study of the subject ‘English as a Foreign Language’), (ii) the questionnaires, now in Chinese Mandarin, were back-translated to English by a staff at one of the Taiwanese universities (Note: the staff teaches ‘English as a Foreign Language’) and another author of this article, who is also a native speaker of both English and Chinese Mandarin, and (iii) cross-checking was made with the English-Chinese Mandarin translation and the Chinese Mandarin-English translation, in total, to ensure consistency and accuracy with the original scales.

### Instruments

We used existing Likert-scale inventories to measure and assess the mentioned concepts. For consistency, we structured the subscales to consist of five ratings: 1 (Completely Disagree) to 5 (Completely Agree). Furthermore, in this section, we report on the results of the psychometric properties of the six scales. Confirmatory factor analysis (CFA) techniques [14, 15] were used to explore the factorial structure of each scale. Specifically, we performed a one-factor congeneric model to determine the appropriateness of the factor loadings of items of each scale. To determine the goodness-of-fit of each congeneric model, we used the threshold values of the following goodness-of-fit indexes: the χ^2^/df ratio, the Comparative Fit Index (CFI)(i.e., CFI value > .95), the Tucker Lewis Index (TLI)(i.e., TLI value > .95), the Root Mean Square Error of Approximation (RMSEA)(i.e., RMSEA value < .07), and the Standardized Root Mean Square Residual (SRMR)(i.e., SRMR value < .05).

#### Realistic best practice

We adapted from the Optimal Outcome Questionnaire [70] and developed five items to measure and assess the concept of RBP [2]. The five items included, for example: “I am content with what I have accomplished so far at this university” and “I can achieve what is being asked of me at this university”. A one-factor congeneric model analysis of this model, Model M_1_, showed a moderate fit, as indicated by the following: χ^2^/df = 12.31, *p* < .001, CFI = .94, TLI = .87, RMSEA = .11 (Lo90 = .08, Hi90 = .13), *p* < .001, and SRMR = .04. We respecified this *a priori* model with the inclusion of an error variance between Item 4 and Item 5. The goodness-of-fit index values for this *a posteriori* model, Model M_2_, showed an improvement in model fit: χ^2^/df = 8.68, *p* < .001, CFI = .96, TLI = .91, RMSEA = .09 (Lo90 = .06, Hi90 = .12), *p* < .01, and SRMR = .03. The Δχ^2^ test between the two models was statistically significant, *p* < .001 (i.e., Δχ^2^_(Model M1 –Model M2)_ = 26.81), indicating support for the *a posteriori* model. To improve the fit further, we respecified Model M_2_ with the inclusion of an error variance between Item 3 and Item 4. The goodness-of-fit index values for this modified model, Model M_3_, improved over that of Model M_2_: χ^2^/df = 7.46, *p* < .001, CFI = .98, TLI = .94, RMSEA = .07 (Lo90 = .05, Hi90 = .09), *p* < .05, and SRMR = .02. The Δχ^2^ test between the two models was statistically significant, *p* < .001 (i.e., Δχ^2^_(Model M1 –Model M2)_ = 12.34), indicating support for the *a posteriori* model. The factor loadings for the five items to the ‘Realistic’ latent variable ranged from .50 to .81 (Mn = .63, SD = .14). Reliability estimate for the scale was .81.

#### Optimal best practice

Similar to that of RBP, we used a shorter version of the Optimal Outcome Questionnaire [70] to measure and assess the concept of OBP [2]. The five items included, for example: “I can achieve much more at university than I have indicated through my work so far” and “I want to learn and do more at university”. The goodness-of-fit index values of this model, Model M_1_, showed a relatively poor fit, as indicated by the following: χ^2^/df = 17.35, *p* > .05, CFI = .80, TLI = .60, RMSEA = .13 (Lo90 = .11, Hi90 = .15), *p* < .001, and SRMR = .07. We respecified this *a priori* model with the inclusion of an error variance between Item 2 and Item 4. The goodness-of-fit index values for this *a posteriori* model, Model M_2_, showed an improvement in model fit: χ^2^/df = 8.06, *p* < .001, CFI = .93, TLI = .83, RMSEA = .08 (Lo90 = .06, Hi90 = .11), *p* < .05, and SRMR = .04. The Δχ^2^ test between the two models was statistically significant, *p* < .001 (i.e., Δχ^2^_(Model M1 –Model M2)_ = 54.48), indicating support for the *a posteriori* model. To improve the fit further, we respecified Model M_2_ with the inclusion of an error variance between Item 2 and Item 3. The goodness-of-fit index values for this modified model, Model M_3_, improved over that of Model M_2_: χ^2^/df = 4.92, *p* < .01, CFI = .97, TLI = .91, RMSEA = .06 (Lo90 = .03, Hi90 = .09), *p* > .05, and SRMR = .03. The Δχ^2^ test between the two models was statistically significant, *p* < .001 (i.e., Δχ^2^_(Model M1 –Model M2)_ = 17.50), indicating support for the *a posteriori* model. The factor loadings for the five items to the ‘Optimal’ latent variable ranged from .63 to .75 (Mn = .69, SD = .06). Reliability estimate for the scale was .79.

#### Personal resolve

We used five items [50] to measure and assess the concept of personal resolve. The items included, for example: “I will do whatever it takes to master my academic studies at university” and “I have a strong desire to succeed in my academic studies at university”. The goodness-of-fit index values showed a good model fit for this model, Model M_1_: χ^2^/df = 7.32, *p* < .001, CFI = .98, TLI = .97, RMSEA = .07 (Lo90 = .05, Hi90 = .09), *p* < .05, and SRMR = .02. The factor loadings for the five items to the ‘Personal Resolve’ latent variable ranged from .60 to .78 (Mn = .74, SD = .07). Reliability estimate for the scale was .85.

#### Motivation towards academic learning

We adapted and used five items from the LOSO Questionnaire [45] to measure and assess the concept of motivation towards academic learning. The items included, for example: “I really try my best at university” and “I always look forward to learning new things at university”. A one-factor congeneric model was moderate in model fit, for example, as indicated from the goodness-of-fit index values: χ^2^/df = 6.61, *p* < .001, CFI = .93, TLI = .87, RMSEA = .08 (Lo90 = .05, Hi90 = .10), *p* < .05, and SRMR = .04. An improvement in model fit was made with the inclusion of an error variance between Item 1 and Item 2. The goodness-of-fit index values for this model, Model M_2_, improved over that of Model M_1_’s: χ^2^/df = 1.68, *p* > .05, CFI = .99, TLI = .98, RMSEA = .03 (Lo90 = .01, Hi90 = .06), *p* > .05, and SRMR = .02. Furthermore, a comparison of the two models, using the Δχ^2^ test (Δχ^2^_(Model M1 –Model M2)_ = 26.35), showed support for Model M_2_. The factor loadings for the five items to the ‘Motivation’ latent variable ranged from .60 to .78 (Mn = .69, SD = .07). Reliability estimate for the scale was .77.

#### Personal well-being experience

We adapted five items from the Academic Well-Being Experience Questionnaire (SWBEQ)[71] to measure and assess the concept of personal well-being experience. The items included, for example: “I find it easy to be yourself at university” and “I find that there are opportunities at university for me to excel”. The goodness-of-fit index values showed a good model fit for this model, Model M_1_: χ^2^/df = 4.18, *p* < .001, CFI = .97, TLI = .95, RMSEA = .06 (Lo90 = .03, Hi90 = .08), *p* > .05, and SRMR = .03. The factor loadings for the five items to the ‘Well-Being’ latent variable ranged from .50 to .68 (Mn = .61, SD = .07). Reliability estimate for the scale was .71.

#### Social relationships

We adapted and used five items from the LOSO Questionnaire [45] to measure and assess the concept of social relationship and being able to relate to others at university. The items included, for example: “I find it easy to relate to others at university” and “I find it difficult to express my feelings to others at university”. A one-factor congeneric model was moderate in model fit, for example, as indicated from the goodness-of-fit index values: χ^2^/df = 9.20, *p* < .01, CFI = .95, TLI = .90, RMSEA = .09 (Lo90 = .07, Hi90 = .12), *p* < .01, and SRMR = .04. An improvement in model fit was made with the inclusion of an error variance between Item 1 and Item 4. The goodness-of-fit index values for this model, Model M_2_, improved over that of Model M_1_’s: χ^2^/df = 4.03, *p* < .01, CFI = .99, TLI = .96, RMSEA = .06 (Lo90 = .03, Hi90 = .08), *p* > .05, and SRMR = .02. Furthermore, a comparison of the two models, using the Δχ^2^ test (Δχ^2^_(Model M1 –Model M2)_ = 29.88), showed support for Model M_2_. The factor loadings for the five items to the ‘Social Relationships’ latent variable ranged from .55 to .80 (Mn = .64, SD = .10). Reliability estimate for the scale was .74.

#### Academic striving

We adapted five items from the Academic Well-Being Experience Questionnaire (SWBEQ)[71] to measure and assess the concept of striving. The items included, for example: “I always strive to achieve good academic results at university” and “I see very little point in achieving high results at university”. A one-factor congeneric model was relatively poor in model fit, for example, as indicated from the goodness-of-fit index values: χ^2^/df = 41.14, *p* < .001, CFI = .85, TLI = .70, RMSEA = .20 (Lo90 = .18, Hi90 = .22), *p* < .001, and SRMR = .07. An improvement in model fit was made with the inclusion of an error variance between Item 2 and Item 4. The goodness-of-fit index values for this model, Model M_2_, improved over that of Model M_1_’s: χ^2^/df = 18.91, *p* < .01, CFI = .95 TLI = .86, RMSEA = .13 (Lo90 = .11, Hi90 = .16), *p* < .001, and SRMR = .04. A comparison of the two models, using the Δχ^2^ test (Δχ^2^_(Model M1 –Model M2)_ = 130.08), showed support for Model M_2_. An additional error variance between Item 1 and Item 3 was included for respecification, which consequently showed an improvement in model fit. The goodness-of-fit index values for this model (i.e., χ^2^/df = 8.12, *p* < .01, CFI = .98, TLI = .95, RMSEA = .08 (Lo90 = .06, Hi90 = .12), *p* < .05, and SRMR = .04), Model M_3_, as well as the Δχ^2^ test (Δχ^2^_(Model M2 –Model M3)_ = 51.31) affirmed the acceptance of this respecification. Finally, for Model M_4_, we included an error variance between Item 3 and Item 5. This respecification, again, improved the model fit (e.g., χ^2^/df = 3.52, *p* < .05, CFI = .98 TLI = .95, RMSEA = .05 (Lo90 = .01, Hi90 = .09), *p* > .0; Δχ^2^_(Model M3 –Model M4)_ = 17.28). The factor loadings for the five items to the ‘Striving’ latent variable ranged from .50 to .80 (Mn = .65, SD = .13). Reliability estimate for the scale was .78.

#### Self-Efficacy for academic learning

We adapted five items from the Motivated Strategies for Learning Questionnaire (MSLQ) [72] to measure and assess self-efficacy beliefs for academic learning. The items included, for example: “I believe I will receive excellent grades at university” and “I'm certain I can master the skills being taught to me at university”. “I have what I need at university to succeed in my academic studies”. The goodness-of-fit index values showed a good model fit for this model, Model M_1_: χ^2^/df = 2.81, *p* < .05, CFI = .99, TLI = .97, RMSEA = .04 (Lo90 = .02, Hi90 = .07), *p* > .05, and SRMR = .02. The factor loadings for the five items to the ‘Self-Efficacy’ latent variable ranged from .50 to .74 (Mn = .62, SD = .09). Reliability estimate for the scale was .72.

## Data analyses

As previously indicated, we used SEM techniques [14, 15] to test the hypothesized *a priori* model, as shown in Fig 1. The significance of this *a priori* model, aside from its correlational nature between L_1_ and L_2_, lies in its examination of potential mediating roles of the three different types of agencies. We conceptualize that in the absence of more complex methodological designs (e.g., the use of experimental design), the use of statistical inference of mediating effects (e.g., L_1_ → personal resolve → L_2_) could in fact offer an alternative ‘proxy’ indicator of optimization. Consequently, unlike other multivariate techniques, SEM is advantageous for permitting us to decompose the total effects into both direct and indirect effects. Moreover, with the assistance of M*Plus* 8.0 [73], it is also possible for us to decompose the indirect effects and consider potential mediating mechanisms [74–76].

The uniqueness of SEM techniques also expand to incorporate errors for measured indicators (i.e., E ≠ 0), and to take into consideration both measurement and structural models [14, 15, 76]. At the same time, SEM is relatively innovative for allowing researchers to test and compare competing *a priori* and *a posteriori* models. At the same time, too, unlike exploratory factor analyses say, SEM (e.g., the use of confirmatory factor analysis) enables the respecification of an *a priori* model and testing the appropriateness of an alternative *a posteriori* model. As indicated in the preceding sections, the extent to which an *a priori* model or an *a posteriori* model is well fitted is determined by the use of various goodness-of-fit index values (e.g., the χ^2^/df ratio).

We used the covariance matrices and maximum likelihood (ML) procedures to test the original hypothesized model. We analysed covariance matrices because correlation matrix analysis is known to have problems, such as producing incorrect goodness-of-fit measures and standard errors [77, 78]. Furthermore, depending on the multivariate normality of the data, we selected to use one of the two estimation procedures–ML or robust ML (RML) procedures. The ML procedure, for example, has been observed to perform reasonably well when data are normally distributed [79].

### SEM analyses: Comparison of competing models

In the initial stage of our SEM analyses, we performed a data screening test by focusing on various multivariate facets–for example, the identification of visible outliers and examination of kurtosis and skewness values (e.g., the values are within the range of ± 1.00). This initial process enabled us to undertake a comparison: compare a baseline model, which we coined as Model M_0_, with the original hypothesized model, which we coined as Model M_1_. Model M_0_, in this case, consisted of the following paths: (i) S to L_1_, (ii) L_1_ to the three As, (iii) the three As to L_2_, (iv) L_1_ to L_2_, and (v) L_2_ to Os (Note: S = motivation towards learning, L_1_ = realistic best practice, L_2_ = optimal best practice, A = personal resolve, social relationship, and self-efficacy, O = academic striving, and personal well-being experience). Relatively simple, the nature of Model M_0_ does not permit the testing and identification of potential mediating mechanisms of L_1_, As, and/or L_2_. For example, the indirect effect of motivation toward learning to personal resolve, via realistic best practice was not determined, consequently because of the absence of the direct structural path from motivation towards learning to personal resolve. The goodness-of-fit index values showed a poor model fit, for example: χ^2^/df = 3.19, *p* < .001, CFI = .87, TLI = .86, RMSEA = .05 (Lo90 = .04, Hi90 = .05), *p* > .05, and SRMR = .06. This poor model fit, from our point of view, is not unexpected given that it is somewhat constrained. The more parsimonious model would consist of the freeing of other structural paths, which we considered in Model M_1_.

Model M_1_, an expansion of Model M_0_, consisted of the additional structural paths: (i) S to As, (ii) S to L_2_, (iii) S to O, (iv) L_1_ to O, and (v) A to O. This model, which is accordance with Baron and Kenny’s [80] criteria for mediating analyses, enabled us to consider the central mediating roles of L_1_, As, and L_2_. Hence, as an example, Model M_1_ would allow us to explore the indirect effect of social relationship on personal well-being experience, mediated in this case by optimal best practice (i.e., there are three paths for examination: social relationship → optimal best practice, optimal best practice → personal well-being experience, and social relationship → personal well-being experience). The goodness-of-fit index values of Model M_1_ showed an improvement in model fit over that of Model M_0_, as indicated by the following: χ^2^/df = 2.77, *p* < .001, CFI = .90, TLI = .90, RMSEA = .04 (Lo90 = .04, Hi90 = .04), *p* > .05, and SRMR = .043. In a similar vein, a Δχ^2^ test supported the respecification of Model M_0_ –Δχ^2^_(Model M0 –Model M1)_ = 341.60 (Δdf = 14), p < .001 [81]. On examination, we contend that the goodness-of-fit values are relatively modest and not that optimal, which partially arose because of the complexity of the *a posteriori* model (e.g., 40 factor loadings). However, having said this, we recognize that this model, Model M_1_, may form the basis for replication and/or advancement in future research.

**Fig 3:**
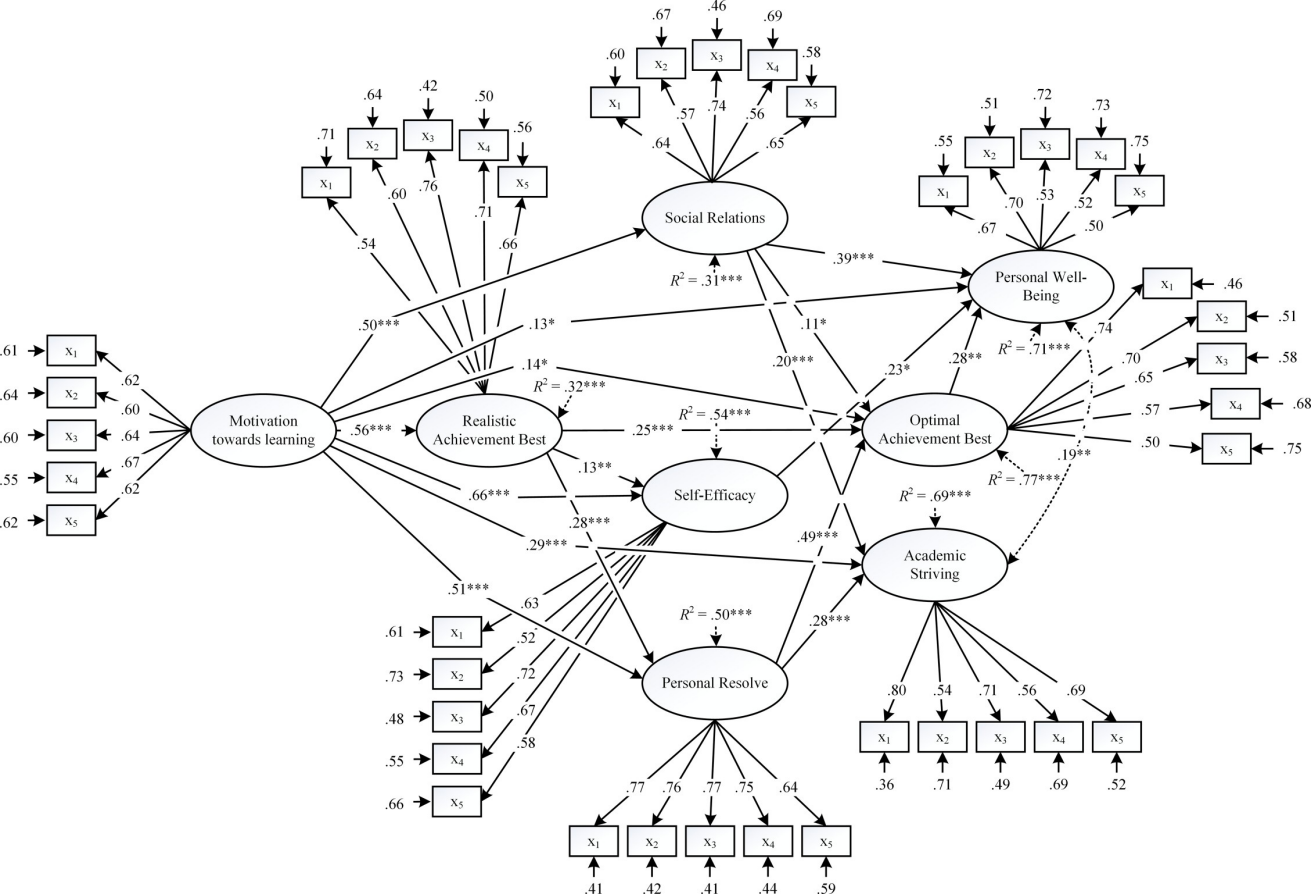
Final solution for model M_1_. Note: * *p* < .05, ** *p* < .01, *** *p* < .001.

Considering Model M_1_, which we highlight the solution in Fig 3, it is interesting to note that of the 24 direct structural paths, 17 were statistically significant. The decomposition of the total effects, as shown in Table 1, also shows that nine indirect effects and 19 total effects were statistically significant. In terms of S, L_1_ and L_2_, As and Os, the direct statistically significant paths emphasize: (i) motivation towards learning as an antecedent of realistic best practice (β = .56, *p* < .001), optimal best practice (β = .14, *p* < .05), different types of agencies (β values ranged from .50 –.66, *p* < .001), and adaptive outcomes (β values ranged from .13, *p* < .05 –.29, *p* < .001), (ii) the impact of realistic best practice on optimal best practice (β = .25, *p* < .001), (iii) social relationship (β = .11, *p* < .05) and personal resolve (β = .49, *p* < .001) as predictors of optimal best practice, (iv) social relationship as a predictor of personal well-being (β = .39, *p* < .001) and academic striving (β = .20, *p* < .05), self-efficacy as a predictor of personal well-being (β = .23, *p* < .05), and personal resolve as a predictor of academic striving (β = .28, *p* < .001), and (v) the impact of optimal best practice on personal well-being (β = .28, *p* < .01).

**Table 1:** Decomposition of direct, indirect and total effects.

	Direct		Indirect		Total	
Realistic Achievement Best						
■ Motivation towards learning	.56	***	-		.56	***
Social Relationship						
■ Realistic Achievement Best	.09		-		.09	
■ Motivation towards learning	.50	***	.05		.55	***
Self-Efficacy						
■ Realistic Achievement Best	.13	*	-		.13	**
■ Motivation towards learning	.66	***	.07	**	.73	***
Personal Resolve						
■ Realistic Achievement Best	.28	***	-		.28	***
■ Motivation towards learning	.51	***	.16	***	.67	***
Personal Well-Being						
■ Optimal	.28	**	-		.28	**
■ Social Relationship	.39	***	.03		.42	***
■ Self-Efficacy	.25	*	.02		.23	*
■ Personal Resolve	-.04		.13	*	.09	
■ Realistic Achievement Best	-.02		.17	***	.15	**
■ Motivation towards learning	.13	*	.54	***	.67	***
Optimal Achievement Best						
■ Social Relationship	.11	*	-		.11	*
■ Self-Efficacy	.05		-		.05	
■ Personal Resolve	.49	***	-		.49	***
■ Realistic Achievement Best	.25	***	.16	***	.41	***
■ Motivation towards learning	.14	*	.57	***	.71	***
Academic Striving						
■ Optimal	.06		-		.06	
■ Social Relationship	.20	***	.01		.21	***
■ Self-Efficacy	.12		.00		.12	
■ Personal Resolve	.28	***	.03		.31	***
■ Realistic Achievement Best	.03		.14	***	.17	***
■ Motivation towards learning	.29	***	.45	***	.73	***

Note:

* *p* < .05

** *p* < .01

*** *p* < .001.

The decomposition of the indirect effects, shown in Table 2, also provided a basis for us to consider mediating analyses, which we report in Table 3. In conjunction with Fig 3 and the results in Table 3, we note the following patterns:

- The indirect effect of motivation towards learning on personal resolve (β = .16, *p* < .001) and self-efficacy (β = .07, *p* < .01), mediated by realistic best practice.
- The indirect effect of motivation towards learning on personal well-being, mediated by realistic best practice and then optimal best practice (β = .04, *p* < .05), and the indirect effect of motivation towards learning on personal well-being, mediated by realistic best practice, personal resolve, and then optimal best practice (β = .02, *p* < .05).
- The indirect effect of motivation towards learning on personal well-being, mediated by personal resolve and then optimal best practice (β = .07, *p* < .05).
- The indirect effect of motivation towards learning on personal well-being, mediated by social relationship (β = .19, *p* < .001).
- The indirect effect of motivation towards learning on personal well-being, mediated by self-efficacy (β = .15, *p* < .05).
- The indirect effect of motivation towards learning on personal well-being, mediated by optimal best practice (β = .20, *p* < .01).
- The indirect effect of motivation towards learning on optimal best practice, mediated by realistic best practice (β = .14, *p* < .001), and the indirect effect of motivation towards learning on optimal best practice, mediated by realistic best practice and then personal resolve (β = .08, *p* < .001).
- The indirect effect of motivation towards learning on optimal best practice, mediated by personal resolve (β = .25, *p* < .001).
- The indirect effect of motivation towards learning on optimal best practice, mediated social relationship (β = .05, *p* < .05).
- The indirect effect of motivation towards learning on academic striving, mediated by realistic best practice and then personal resolve (β = .05, *p* < .01).
- The indirect effect of motivation towards learning on academic striving, mediated by personal resolve (β = .15, *p* < .001), and the indirect effect of motivation towards learning on academic striving, mediated by realistic best practice and then personal resolve (β = .05, *p* < .01).
- The indirect effect of motivation towards learning on academic striving, mediated by social relationship (β = .10, *p* < .01).
- The indirect effect of realistic best practice on personal well-being, mediated by optimal best practice (β = .07, *p* < .05), and the indirect effect of realistic best practice on personal well-being, mediated by personal resolve and then optimal best practice (β = .04, *p* < .05).
- The indirect effect of realistic best practice on optimal best practice, mediated by personal resolve (β = .14, *p* < .001).
- The indirect effect of realistic best practice on academic striving, mediated by personal resolve (β = .08, *p* < .01).
- The indirect effect of personal resolve on personal well-being, mediated by optimal best practice (β = .13, *p* < .01).

Overall, from this detailed summary, we note that realistic best practice, the three types of agencies, and optimal best practice functioned as potential mediators between motivation towards learning and personal well-being and academic striving. What is of interest too, from the decomposition of indirect effects, is the combined sequencing of realistic best practice, the three types of agencies, and optimal best practice–for example: the combined sequencing of personal resolve and optimal best practice to mediate the effect of motivation towards learning on personal well-being. This evidence, overall, accentuates the triarchic anchor of realistic best practice, the three different types of agencies, and optimal best practice.

**Table 2:** Decomposition of indirect effects.

Predictor					Outcome	*β*	*p*
Motivation	Realistic				Resolve	.16	***
Motivation	Realistic				Self-Efficacy	.07	**
Motivation	Realistic				Social Relationship	.05	
Resolve	Optimal				Personal Well-Being	.13	*
Pathways	Optimal				Personal Well-Being	.02	
Social Relationship	Optimal				Personal Well-Bring	.03	
Realistic	Optimal				Personal Well-Being	.07	*
Realistic	Resolve				Personal Well-Being	-.01	
Realistic	Self-Efficacy				Personal Well-Being	.03	
Realistic	Social Relationship				Personal Well-Being	.03	
Realistic	Resolve		Optimal		Personal Well-Being	.04	*
Realistic	Self-Efficacy		Optimal		Personal Well-Being	.00	
Realistic	Social Relationship		Optimal		Personal Well-Being	.00	
Motivation	Realistic				Personal Well-Being	-.00	
Motivation	Optimal				Personal Well-Being	.04	
Motivation	Resolve				Personal Well-Being	-.02	
Motivation	Self-Efficacy				Personal Well-Being	.15	*
Motivation	Social Relationship				Personal Well-Being	.19	***
Motivation	Realistic		Optimal		Personal Well-Being	.04	*
Motivation	Resolve		Optimal		Personal Well-Being	.07	*
Motivation	Self-Efficacy		Optimal		Personal Well-Being	.01	
Motivation	Social Relationship		Optimal		Personal Well-Being	.02	
Motivation	Realistic		Resolve		Personal Well-Being	-.01	
Motivation	Realistic		Self-Efficacy		Personal Well-Being	.02	
Motivation	Realistic		Social Relationship		Personal Well-Being	.02	
Motivation	Realistic	Resolve		Optimal	Personal Well-Being	.02	*
Motivation	Realistic	Self-Efficacy		Optimal	Personal Well-Being	.00	
Motivation	Realistic	Social Relationship		Optimal	Personal Well-Being	.00	
Realistic	Resolve				Optimal	.14	***
Realistic	Self-Efficacy				Optimal	.01	
Realistic	Social Relationship				Optimal	.01	
Motivation	Realistic				Optimal	.14	***
Motivation	Resolve				Optimal	.25	***
Motivation	Self-Efficacy				Optimal	.04	
Motivation	Social Relationship				Optimal	.05	*
Motivation	Realistic		Resolve		Optimal	.08	***
Motivation	Realistic		Self-Efficacy		Optimal	.00	
Motivation	Realistic		Social Relationship		Optimal	.01	
Realistic	Optimal				Academic Striving	.02	
Realistic	Resolve				Academic Striving	.08	***
Realistic	Self-Efficacy				Academic Striving	.02	
Realistic	Social Relationship				Academic Striving	.02	
Realistic	Resolve		Optimal		Academic Striving	.01	
Realistic	Self-Efficacy		Optimal		Academic Striving	.00	
Realistic	Social Relationship		Optimal		Academic Striving	.00	
Motivation	Realistic				Academic Striving	.02	
Motivation	Optimal				Academic Striving	.01	
Motivation	Resolve				Academic Striving	.15	***
Motivation	Self-Efficacy				Academic Striving	.08	
Motivation	Social Relationship				Academic Striving	.10	***
Motivation	Realistic		Optimal		Academic Striving	.01	
Motivation	Resolve		Optimal		Academic Striving	.02	
Motivation	Self-Efficacy		Optimal		Academic Striving	.00	
Motivation	Social Relationship		Optimal		Academic Striving	.00	
Motivation	Realistic		Resolve		Academic Striving	.05	**
Motivation	Realistic		Self-Efficacy		Academic Striving	.01	
Motivation	Realistic		Social Relationship		Academic Striving	.01	
Motivation	Realistic	Resolve		Optimal	Academic Striving	.01	
Motivation	Realistic	Self-Efficacy		Optimal	Academic Striving	.00	
Motivation	Realistic	Social Relationship		Optimal	Academic Striving	.00	
Resolve	Optimal				Academic Striving	.03	
Self-Efficacy	Optimal				Academic Striving	.00	
Social Relationship	Optimal				Academic Striving	.01	

Note:

* *p* < .05

** *p* < .01

*** *p* < .001. Motivation = motivation towards learning, Realistic = realistic achievement best, Optimal = optimal achievement best, Resolve = personal resolve

**Table 3:** Mediating effects.

Predictor		Mediator			Outcome	*β*	*p*
Motivation		Realistic			Resolve	.16	***
Motivation		Realistic			Self-Efficacy	.07	**
Motivation		Realistic			Social Relationship	.05	
Motivation		Realistic			Personal Well-Being	.08	**
	Motivation	Realistic			Personal Well-Being	-.01	
	Motivation	Realistic		Optimal	Personal Well-Bring	.04	*
	Motivation	Realistic		Resolve	Personal Well-Being	-.01	
	Motivation	Realistic		Self-Efficacy	Personal Well-Being	.02	
	Motivation	Realistic		Social Relationship	Personal Well-Being	.02	
	Motivation	Realistic	Resolve	Optimal	Personal Well-Being	.02	*
	Motivation	Realistic	Self-Efficacy	Optimal	Personal Well-Being	.00	
	Motivation	Realistic	Social Relationship	Optimal	Personal Well-Being	.00	
Motivation		Resolve			Personal Well-Being	.07	
	Motivation	Resolve			Personal Well-Bring	-.02	
	Motivation	Resolve		Optimal	Personal Well-Being	.07	*
	Motivation	Realistic		Resolve	Personal Well-Being	-.01	
	Motivation	Realistic	Resolve	Optimal	Personal Well-Being	.02	*
Motivation		Social Relationship			Personal Well-Being	.23	***
	Motivation	Social Relationship			Personal Well-Being	.19	***
	Motivation	Social Relationship		Optimal	Personal Well-Being	.02	
	Motivation	Realistic		Social Relationship	Personal Well-Being	.02	
	Motivation	Realistic	Social Relationship	Optimal	Personal Well-Being	.00	
Motivation		Self-Efficacy			Personal Well-Being	.18	**
	Motivation	Self-Efficacy			Personal Well-Being	.15	*
	Motivation	Self-Efficacy		Optimal	Personal Well-Being	.01	
	Motivation	Realistic		Self-Efficacy	Personal Well-Being	.02	
	Motivation	Realistic	Self-Efficacy	Optimal	Personal Well-Being	.00	
Motivation		Optimal			Personal Well-Being	.20	**
Realistic		Resolve			Personal Well-Being	.03	
	Realistic	Resolve			Personal Well-Being	-.01	
	Realistic	Resolve		Optimal	Personal Well-Being	.04	*
Realistic		Self-Efficacy			Personal Well-Being	.03	
	Realistic	Self-Efficacy			Personal Well-Being	.03	
	Realistic	Self-Efficacy		Optimal	Personal Well-Being	.00	
Realistic		Social Relationship			Personal Well-Being	.04	
	Realistic	Social Relationship			Personal Well-Being	.03	
	Realistic	Social Relationship		Optimal	Personal Well-Being	.01	
Relating		Optimal			Personal Well-Being	.03	
Self-Efficacy		Optimal			Personal Well-Being	.02	
Resolve		Optimal			Personal Well-Being	.13	**
Realistic		Optimal			Personal Well-Being	.11	*
	Realistic	Optimal			Personal Well-Being	.07	*
	Realistic	Resolve		Optimal	Personal Well-Being	.04	*
	Realistic	Self-Efficacy		Optimal	Personal Well-Being	.00	
	Realistic	Social Relationship		Optimal	Personal Well-Being	.00	
Motivation		Realistic			Optimal	.23	***
	Motivation	Realistic			Optimal	.14	***
	Motivation	Realistic		Resolve	Optimal	.08	***
	Motivation	Realistic		Self-Efficacy	Optimal	.00	
	Motivation	Realistic		Social Relationship	Optimal	.01	
Motivation		Resolve			Optimal	.33	***
	Motivation	Resolve			Optimal	.25	***
	Motivation	Realistic		Resolve	Optimal	.08	***
Motivation		Social Relationship			Optimal	.06	*
	Motivation	Social Relationship			Optimal	.05	*
	Motivation	Realistic		Social Relationship	Optimal	.01	
Motivation		Self-Efficacy			Optimal	.04	
	Motivation	Self-Efficacy			Optimal	.04	
	Motivation	Realistic		Self-Efficacy	Optimal	.00	
Realistic		Resolve			Optimal	.14	***
Realistic		Self-Efficacy			Optimal	.01	
Realistic		Social Relationship			Optimal	.01	
Motivation		Realistic			Academic Striving	.10	***
	Motivation	Realistic			Academic Striving	.02	
	Motivation	Realistic		Optimal	Academic Striving	.01	
	Motivation	Realistic		Resolve	Academic Striving	.05	**
	Motivation	Realistic		Self-Efficacy	Academic Striving	.01	
	Motivation	Realistic		Social Relationship	Academic Striving	.01	
	Motivation	Realistic	Resolve	Optimal	Academic Striving	.01	
	Motivation	Realistic	Self-Efficacy	Optimal	Academic Striving	.00	
	Motivation	Realistic	Social Relationship	Optimal	Academic Striving	.00	
Motivation		Resolve			Academic Striving	.21	***
	Motivation	Resolve			Academic Striving	.15	***
	Motivation	Resolve		Optimal	Academic Striving	.02	
	Motivation	Realistic		Resolve	Academic Striving	.05	**
	Motivation	Realistic	Resolve	Optimal	Academic Striving	.01	
Motivation		Social Relationship			Academic Striving	.11	***
	Motivation	Social Relationship			Academic Striving	.10	***
	Motivation	Social Relationship		Optimal	Academic Striving	.00	
	Motivation	Realistic		Social Relationship	Academic Striving	.01	
	Motivation	Realistic	Social Relationship	Optimal	Academic Striving	.00	
Motivation		Self-Efficacy			Academic Striving	.09	
	Motivation	Self-Efficacy			Academic Striving	.08	
	Motivation	Self-Efficacy		Optimal	Academic Striving	.00	
	Motivation	Realistic		Self-Efficacy	Academic Striving	.01	
	Motivation	Realistic	Self-Efficacy	Optimal	Academic Striving	.00	
Realistic		Resolve			Academic Striving	.09	***
	Realistic	Resolve			Academic Striving	.08	**
		Resolve		Optimal	Academic Striving	.01	
Realistic		Self-Efficacy			Academic Striving	.02	
	Realistic	Self-Efficacy			Academic Striving	.02	
	Realistic	Self-Efficacy		Optimal	Academic Striving	.00	
Realistic		Social Relationship			Academic Striving	.02	
	Realistic	Self-Efficacy			Academic Striving	.02	
	Realistic	Social Relationship		Optimal	Academic Striving	.00	
Social Relationship		Optimal			Academic Striving	.01	
Self-Efficacy		Optimal			Academic Striving	.00	
Resolve		Optimal			Academic Striving	.03	
Realistic		Optimal			Academic Striving	.03	
	Realistic	Optimal			Academic Striving	.02	
	Realistic	Resolve		Optimal	Academic Striving	.01	
	Realistic	Self-Efficacy		Optimal	Academic Striving	.00	
	Realistic	Social Relationship		Optimal	Academic Striving	.00	
Motivation		Optimal			Academic Striving	.04	
	Motivation	Optimal			Academic Striving	.01	
	Motivation	Realistic		Optimal	Academic Striving	.01	
	Motivation	Resolve		Optimal	Academic Striving	.02	
	Motivation	Self-Efficacy		Optimal	Academic Striving	.00	
	Motivation	Social Relationship		Optimal	Academic Striving	.00	
	Motivation	Realistic	Resolve	Optimal	Academic Striving	.01	
	Motivation	Realistic	Self-Efficacy	Optimal	Academic Striving	.00	
	Motivation	Realistic	Social Relationship	Optimal	Academic Striving	.00	

Note:

* *p* < .05

** *p* < .01

*** *p* < .001.

## Discussion of results

Optimal best practice, reflecting the paradigm of positive psychology [8, 9], is a central facet of human agency. Experience of optimal best practice indicates personal growth, an internal state of flourishing, mental strength, and persistence. Experience of optimal best practice has wide-ranging implications for individuals and society and, more importantly, indicates enrichment, prosperity, and a positive outlook of life. One notable aspect of understanding of optimal best practice entails its achievement and existing state–that is, how does a person achieve a state of optimal best? The present study, correlational in nature, addresses this question and seeks clarity and understanding of how a state of optimal best practice is reached. Our non-experimental investigation, conceptually grounded in the study of optimization [1, 11], has produced clear evidence, which attests to both significant theoretical and methodological contributions for further development.

### Clarity and understanding of optimal best practice

In recent years, researchers have focused on the paradigm of positive psychology and education [13, 35] to explain the essence and meaning of the proactivity of human agency, which in this case emphasizes the importance of autonomy, enrichment, proactivity, and engagement. Positive psychology, as an individual branch of psychology, differs from the traditional deficit theoretical models of cognition, emotional functioning, and human behaviour. The uniqueness of positive psychology, we contend, lies in its nature and characteristics to direct a person to experience the well-meaning and enrichment of life. This theoretical positioning, we reason, acknowledges a person’s intention to progress, to enjoy life, and to embrace future outlooks with a sense of optimism, motivation, resolute, and purpose. From this understanding, we postulate that the nature of optimal best practice and, in particular, the process of optimization [1, 2] could complement and enhance our understanding of the tenets of positive psychology (e.g., personal experience of flourishing)–for example, the achievement of optimal best practice, a topical inquiry that is unclear at present, may indeed reflect a person’s enriched experience of life in a subject matter.

Foremost from our conceptualization is the question of how a state of optimal best practice is reached. There are two possible considerations: relevant sources of information that could serve as antecedents, and different types of agencies that could optimize a person’s learning experience. From our SEM analyses of data situated within a university context, a student’s current level of best practice positively influences his/her optimal best practice. This finding is in accordance with social cognitive theory [49, 59], which places emphasis on the importance of a person’s existing accomplishments in a subject matter–successful accomplishments, in this instance, are more likely to indicate optimism, confidence, and a corresponding level of expectations for future development. Difficulties and failures at present, in contrast, are more analogous with a student’s reporting of a low-to-moderate level of optimal best. Aside from this indicator of existing experiences and accomplishments, we note that a student’s state of motivation towards learning also plays an important role in the determination of his optimal best practice. A high level of motivation towards learning (e.g., I can do much better at university….), in this case, is closely aligned with an exceptional level of best practice (e.g., I can achieve much more in…. at university).

In a similar vein, but more poignant in the matter, we note that two of the three agencies positively influenced optimal best practice: social relationship and personal resolve. Social relationships at university, in this case, may involve a student’s ability to relate to others for the purpose of friendship, social and/or emotional support, etc. This social engagement, as indicated, is effective and may transfer to academic realms [45, 56]. Positive and proactive social relationships may assist students to seek academic assistance from those are more capable. At the same time, reflecting a performance-based approach [82], social relationships (e.g., student-student relationship) may also stimulate a sense of academic competition, and provide normative information for the purpose of personal benchmarking. In the context of the present study, for example, a student may capitalize on his social relationship with friends to assist in the determination and establishment of an appropriate level of best practice in terms of academic learning. The student, in this case, may use his/her close friend’s academic accomplishments as a point of reference and motivation.

Personal resolve, as we detailed, is an interesting concept that closely relates to the process of optimization [1, 50]. As indicated from our SEM analyses, a student’s personal resolve positively influenced his/her optimal best practice. This finding reflects the characteristics of personal resolve, which emphasize the importance of decisiveness, resolute, and the student’s attention to task. In this analysis, we contend that a student’s state of resolute would help to ensure that she remains steadfast and deliberate in a course of action. Moreover, from our point of view, a heightened state of personal resolve would instil a corresponding level of motivation and confidence for academic learning. A lack of personal resolve, in contrast, would indicate a case of indecisiveness, resulting in uncertainty with regard to optimal best practice.

### A case of optimization

One notable inquiry that remains elusive, to date, is the impact and operational nature of the process of optimization [1, 11]. Existing theorizations and conceptualizations posit that optimization, as an underlying process, would serve to ‘optimize’ a person’s L_1_ to that of L_2_. This positive impact of optimization, which we depict as ‘↓’ in the present study, is relatively difficult to measure and assess. The fundamental premise, in this case, is that the impact of optimization (↓) is intricately associated with a positive difference between L_1_ and L_2_. At this stage of research development, we argue that no adequate methodological design is available, and the use of a non-experimental design may offer some limited insights into this process of optimization. In particular, aligning to Baron and Kenny’s [80] criteria, we contend that mediating mechanisms could provide some preliminary insights into the role of optimization. In this case, as shown in Fig 1, we conceptualized that validation of a mediating effect (e.g., L_1_ → personal resolve → L_2_) could in fact offer an alternative proxy indicator of an optimizing effect. On this basis, as reported in Fig 3, personal resolve is noted to mediate the effect of realistic best practice on optimal best practice (β = .14, *p* < .001). This evidence, insightful but relatively limited, details the potentiality for further research development into the ‘methodological mechanisms’ that could elucidate the optimizing role of personal resolve, etc. At the same time, from a theoretical positioning, the potential optimizing role of personal resolve also accentuates its nature and characteristics [1, 50].

The non-statistical mediating roles of social relationship and self-efficacy arise from the non-statistical effect of realistic best practice on social relationship and the non-statistical effect of self-efficacy on optimal best practice, respectively. The statistical significance of personal resolve empirically supports Phan et al.’s [1] Framework of Achievement Bests theorization, especially in relation to its central role. According to the authors’ theorization, personal resolve is an internal sub-process that could serve in a larger cognitive system to optimize a person’s learning and performance outcome. As we alluded, aside from mediating the relationship between realistic best practice and optimal best practice, personal resolve is also statistically significant in its mediating mechanism between motivation towards learning and the two types of adaptive outcomes (e.g., motivation towards learning → personal resolve → academic striving).

### Similarities and differences between realistic and optimal best practice

The nature and characteristics of realistic best practice and optimal best practice are of considerable interest, both theoretically and methodologically [1, 2]. Aside from the importance of time precedence, which differentiates the two levels of best practice, we contend that there are a number of notable similarities and differences. One similarity, as shown in Fig 3, is the positive impact of motivation towards learning on the formulation of both realistic best practice and optimal best practice. A student’s internal state of motive for learning, in this case, serves as an important antecedent of learning and performance outcome. Another similarity, likewise, is concerned with the mediating role of each of the two levels of best practice (e.g., motivation towards learning → realistic best practice → personal resolve, and motivation towards learning → optimal best practice → personal well-being).

A major difference, in contrast, lies in the explanatory and predictive role of each level of best practice: the impact of realistic best practice on self-efficacy and personal resolve (i.e., L_1_ → As) *versus* the impact of optimal best practice on personal well-being (i.e., L_2_ → O). This finding, in fact, discerns the dissimilar characteristics between realistic best practice and optimal best practice. From our conceptualization and subsequent empirical validation, as reported in Table 1 and Fig 3, realistic best practice is noted to act as a potential source of different types of agencies, which could then optimize a student’s learning experience and performance outcome. Optimal best practice, however, is shown to operate as an important predictor of future educational outcomes. This testament is poignant as it emphasizes, perhaps, the motivational nature of optimal best practice–that is, personal experience of optimal best practice may serve as a motivational index of adaptive outcomes for further development.

### Agencies of optimization and adaptive outcomes

From Fig 3 and as reported in Table 1, the three agencies examined positively influenced the two types of adaptive outcomes: social relationship influenced both personal well-being and academic striving, whereas self-efficacy influenced personal well-being and personal resolve influenced academic striving. This finding empirically supports existing theorizations into the saliency of personal resolve [3, 50], social relationship [45, 56], and self-efficacy [49, 61]. Personal resolve, as we mentioned, is a motivational construct that could serve to facilitate the achievement of different types of adaptive outcomes [50]. For example, an interesting aspect of development, and coinciding with the focus on optimal best practice, is that of the achievement of academic striving [40]. Academic striving, similar to the concept of thriving [30, 31], emphasizes the importance of flourishing and a person’s state of positive outlook in life. This proactive mindset, in particular, mobilizes the person’s aspiration and determination to seek successful fulfilment of different realistic endeavours in life (e.g., obtaining scholarly distinction in English literature). Indication of academic striving suggests a case of proactivity, excitement, and intrinsic motivation.

The totality of the social milieu, as previous research has shown, plays an important role in facilitating and shaping a person’s cognitive development [83, 84]. Social relationships in educational settings, as evidence indicates, serve to help students cope and adjust in their schooling experiences [56–58]. Aside from enriched academic experiences, educators and researchers have reported that proactive social relationships may also feature in the enrichment and cultivation of subjective well-being experiences. Subjective well-being, as we previously mentioned, is relatively complex and diverse in scope and coverage [11, 68]. One personal attribute of subjective well-being in educational contexts consists of the extent to which a student feels positive about schooling [45]–for example, does she enjoy attending school? Are there perceived possibilities for the student to excel, academically and/or non-academically? In this analysis, from our point of view, social relationships may provide opportunities for emotional and social support, which could then help students feel safe and positive about the schooling processes, in general. A student, in this case, may seek out friends, peers, and capable others for assistance, friendship, and guidance to improve learning, and/or to explore different academic frontiers.

At the same time, of course, the present study affirmed the importance of the central role of personal self-efficacy [49]. It is interesting to note that academic self-efficacy, measured and assessed at a non-microanalytical level (e.g., “I'm certain I can master the skills being taught to me at university”), is analogously aligned with a corresponding educational outcome that is of a non-task specific nature. This finding affirms the importance of the issue of *contextualization and specificity* of self-efficacy [49, 50, 61], which emphasizes the ‘constructive and close alignment’ between self-judgment of perceived competence and the criterial task under investigation. Personal well-being, from our point of view, is a global entity that espouses different schooling experiences [45, 68]. In their recent longitudinal study, likewise, Phan and colleagues [50] included a comparable concept known as ‘schooling experience’. This mentioning of a student’s schooling experience coincides with our emphasis of personal well-being and contends that, importantly, the process of formal education is not limited to the sole index of academic performance. A healthy personal well-being, reflecting enjoyment of school or university for social reasons (e.g., friendship), fulfilment of personal endeavours (e.g., doing well academically), and/or a perceived sense of belonging, may also indicate successful schooling.

## Caveats and methodological contributions for consideration

As a point of summation, the uniqueness of the present study lies in its quest to address a fundamental question, namely: the seeking of clarity and verification into the process of optimization, which could facilitate and optimize a person’s state of functioning (e.g., cognitive functioning). The present study has empirically substantiated existing theorizations [1, 2, 11] and, importantly, produced evidence that could facilitate further research development. The results reported in Fig 3, as a point of summary, provide a visual depiction of the following: (i) appropriate sources of information that could determine a person’s level of best practice (e.g., realistic best practice), (ii) the ‘proxy’ optimizing nature of different types of agencies (e.g., personal resolve), which could facilitate the achievement of optimal best practice, and (iii) the mediating mechanism of best practice (e.g., realistic best practice) and its predictive effect.

We recognize that there are some notable limitations pertaining to both the present study and previous research investigations [6, 50]. Firstly, as we previously detailed, the overall sample used for the study was convenient and limited us from making generalization to the wider Taiwanese population. By the same token, however, we need to take into consideration the sociocultural contextualization of the inquiry, at hand–meaning, in this sense, that the conclusion drawn from our analyses is contextualized within an Asian learning and sociocultural-historical context, which of course differs from the Western context [3, 85–87]. Taiwanese families, similar to families of other Asian countries, are bounded by personal beliefs of *collectivism* [88, 89] and *filial piety* [86, 87]. Pride, family honour and social recognition, and respect, in this case, form the basis of Taiwanese society, resulting in Taiwanese families and students perceiving and conceptualizing academic achievement differently. From an early age, as we mentioned, Taiwanese students are espoused with the ethos and family pressure to do well at school. Successful schooling, in this sense, is an important hallmark that perpetuates over the course of time, compelling and motivating many Taiwanese students to work hard.

Another limitation relates to the engagement of appropriate methodologies that could enable the measurement and assessment of the nature of optimal best practice and, more importantly, the process of optimization. Our use of cross-sectional, non-experimental data is limited and did not permit a true examination of the intricate process of optimization [1]. In a similar vein, as existing theorizations have shown [90, 91], cross-sectional data that are non-experimental do not provide grounding for statistical analyses of causal inference between variables. At best, we acknowledge that cross-sectional data, as our results have shown, only provide empirical understanding into the associative patterns between different optimizing and adaptive variables. Furthermore, we contend that given the complexity of optimization, the use of non-experimental data is somewhat inadequate. ‘Methodological appropriateness’, which we introduce as a topical theme for discussion, is an inquiry that in itself is worthy of development. In a recent article, we coined a term known as the ‘index of optimization’ (IO), which encompasses a quantified numerical representation of optimization–γ (i.e., optimizing effect)[7]. This proposition, however, also raises an important issue for consideration–namely, the design and development of adequate methodological measures that could validate the numerical value of γ.

We purport that the use of regression modelling and, hence, a regression value (e.g., β = .41, *p* < .05) to define the derivative of γ (i.e., β ≈ γ) is erroneous. An ‘optimizing effect’, in this sense, is more than indication of an association between two variables. We argue that an optimizing effect, from the perspective of optimization [1, 7, 11], espouses the intricacy of the ‘interrelationship’ between the direct impact of an optimizing agent (e.g., personal resolve) and the improvement of L_2_ from L_1_. From this complexity, the use of SEM techniques with non-experimental data does not provide a true capture of the operational nature of optimization. A focus of inquiry then, from our rationalization, is to consider a methodological design that could gauge into the relationship between the operational functioning of different types of agencies and the difference between L_1_ and L_2_.

**Fig 4:**
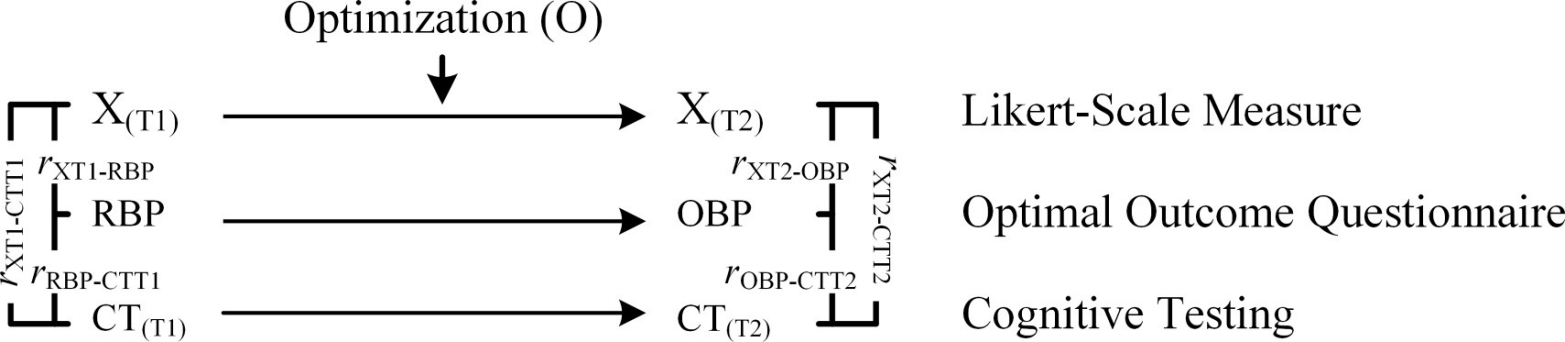
Conceptualization of methodological measurements.

Aside from the preceding mentioning, it is also noteworthy for educators and researchers to focus on the measurement and assessment of both L_1_ and L_2_. At present, in terms of academic cognitive competence, there are three comparable approaches: (i) the use of Optimal Outcome Questionnaire [70] with its two corresponding subscales, (ii) the use of Likert-scale inventories (e.g., the Comprehensive Inventory of Thriving (CIT): [30]), administered to subjects on multiple occasions and calculating their responses and mean score differences (e.g., Δ(_MnT1-MnT2)_, etc.), and (iii) the use of cognitive competence tests (e.g., a high-stake exam), administered to subjects on multiple occasions and calculating their answers and mean score differences. These three methodological measures, from our point of view, are comparable with each other and may provide complementary insights into the measurement and assessment of different levels of best practice. One possible inquiry for consideration, in this analysis, entails the cross-validation of the three quantitative measures, using factorial statistical techniques [92]. This approach, as conceptualized in Fig 4, is innovative and may enable researchers to test and validate the positive associations and construct validity of the three measures.

The study of different levels of best practice, as researchers have indicated [4, 6], is relatively complex, methodologically. We recognize that delving into the nature of best practice requires the precedence of time difference–hence, as shown in Fig 4, a Likert-scale measure (e.g., denoted as ‘X’) and a cognitive test (e.g., denoted as ‘ST’), administered on multiple occasions (i.e., T_1_, T_2_, …T_*n*_), may enable researchers to determine positive or negative score differences. Positive mean score differences, in this case, would indicate achievement of optimal best practice (i.e., a mean score of L_2_ > a mean score of L_1_). The use of the Optimal Outcome Questionnaire [70], we contend, is more valid when it is administered on multiple occasions–for example, the administration of the RBP at T_1_ and the OBO at T_2_. Overall then, from our conceptualization, we postulate that the three methodological measures would positively associate with each other at T_1_, T_2_, etc. (e.g., *r*_XT1-RBP_ denoting the association between X at T_1_ and RBP at T_1_, where *r* = correlation, X = Likert-scale inventory, RBP = Realistic Best Subscale).

## Supporting information

S1 Table (Covariance and correlation matrixes). (RAR)
